# Toluene Removal from Sandy Soils via In Situ Technologies with an Emphasis on Factors Influencing Soil Vapor Extraction

**DOI:** 10.1155/2014/416752

**Published:** 2014-01-23

**Authors:** Mohammad Mehdi Amin, Mohammad Sadegh Hatamipour, Fariborz Momenbeik, Heshmatollah Nourmoradi, Marzieh Farhadkhani, Fazel Mohammadi-Moghadam

**Affiliations:** ^1^Environment Research Center, Isfahan University of Medical Sciences, Isfahan, Iran; ^2^Chemical Engineering Department, Faculty of Engineering, University of Isfahan, Isfahan, Iran; ^3^Department of Chemistry, University of Isfahan, Isfahan, Iran; ^4^Department of Environmental Health Engineering, School of Health, Ilam University of Medical Sciences, Ilam, Iran; ^5^Faculty of Health, Shahr-e-kord University of Medical Sciences, Rahmatieh, P.O. Box 88155-383, Shahr-e-kord, Iran

## Abstract

The integration of bioventing (BV) and soil vapor extraction (SVE) appears to be an effective combination method for soil decontamination. This paper serves two main purposes: it evaluates the effects of soil water content (SWC) and air flow rate on SVE and it investigates the transition regime between BV and SVE for toluene removal from sandy soils. 96 hours after air injection, more than 97% removal efficiency was achieved in all five experiments (carried out for SVE) including 5, 10, and 15% for SWC and 250 and 500 mL/min for air flow rate on SVE. The highest removal efficiency (>99.5%) of toluene was obtained by the combination of BV and SVE (AIBV: Air Injection Bioventing) after 96 h of air injection at a constant flow rate of 250 mL/min. It was found that AIBV has the highest efficiency for toluene removal from sandy soils and can remediate the vadose zone effectively to meet the soil guideline values for protection of groundwater.

## 1. Introduction

There are thousands of underground storage tanks (USTs) in Iran. Gasoline leakage from USTs can contaminate the vadose (unsaturated) zone and finally ground water [1]. Gasoline is a complex mixture of many volatile and semivolatile hydrocarbons including benzene, toluene, ethyl-benzene, and xylene isomers (BTEX) [1–5].

BTEX compounds have many adverse effects on the environment and human health due to their neurotoxic, carcinogenic, and teratogenic properties [6]. Toluene is a well-known constituent of BTEX, which is rapidly absorbed through respiratory and gastrointestinal tracts; the exposure to which results in various symptoms including weakness, headache, vertigo, and ataxia [7].

The United States Environmental Protection Agency (USEPA) has classified toluene compound as priority pollutant [3, 5, 6, 8–11], the guideline value of which in the sandy soil for protection of ground water is 12 mg/kg of the soil [9].

SVE is one of the most common treatment procedures to remove the gasoline compounds from soil with medium to high porous media [8, 12]. The advantages that SVE system holds over the other used technologies are its high decontamination in short term [13, 14], cost-effectiveness [13, 15], simplicity of equipment, system operation, and maintenance [8].

Fischer et al. [16] found out that the SVE can remove toluene with an efficiency of 97% after 25 h extraction in dry soil. Qin et al. [13] reported that the maximum removal efficiency of chlorobenzene via SVE with initial concentration of 1.1 mg/g soil at a gas flow rate of 0.3 m^3^/h was 95% during an 80-hour operation.

Among the in situ treatment methods applied in USA National Super Fund Projects during 1980 to 2000, 26% of these procedures were attributed to SVE [3, 13]. In addition to SVE, bioventing is also one of the standardand cost-competitive techniques [17] for cleaning petroleum-contaminated vadose zone soils [14, 18].

Both the SVE and BV are in situ aeration-based remediation technologies [19],but the major disadvantage of BV is that it is time-consuming [14, 18].

Dupont and Lakshmiprasad [20] concluded that 6.16% 94.62%, and 99.33% of benzene, toluene, and p-xylene, respectively, were removed by BV at 4000 h and 1.0 pore volume/d. BV can be used in combination with other technologies such as SVE. However, SVE can reduce the treatment time needed by BV only [14]. Thus, the increase of airflow through the subsurface supplied by SVE increases the oxygen concentration of vadose zone and enhances the biodegradation of contaminants [13, 15].

Laboratory studies by Magalhães et al. [14] have shown that AIBV is a very efficient technique for soil decontamination. They observed that toluene with initial concentration of 2 and 14 mg/g soil was reduced by 99% within 5 days at a gas flow rate of 0.13 L/min.

The purpose of this laboratory-scale study was to investigate the effects of such factors as air flow rate and SWC on the SVE; the effect of AIBV on toluene removal from sandy soils was also investigated, due to limitation of experimental information on the AIBV in soil.

## 2. Materials and Methods

### 2.1. Sandy Soil Characteristics

**Figure 1:**
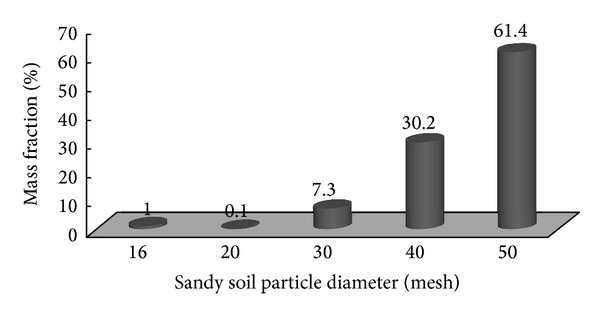
Size distribution of sandy soil.

The sandy soil was taken from a depth of 2 m in the coastline area of Asalouyeh, Iran. It was repeatedly washed with water to get clear water. Then it was dried, first at room temperature during 5 days and after, it was autoclaved at 121°C, 15 psi, for 30 min. The size distribution of soil is shown in Figure 1.

The physicochemical properties of the soil were determined; the results are summarized in Table 1.

### 2.2. Media

The mineral medium (MM) had the following composition [21]: Na_2_HPO_4_·H_2_O (0.134 g/L; Merck, Germany), KH_2_PO_4_ (0.03 g/L; Merck, Germany), NaCl (0.5 g/L; Merck, Germany), NH_4_Cl (3.982 g/L; Scharlau, Spain), MgSO_4_·7H_2_O (2.47 g/100 mL; Merck Germany); 1 mL salt/100 mL medium, CaCl_2_ (111 mg/100 mL; Merck Germany); 1 mL salt/100 mL medium.

Toluene (0.5 g/L; purity of 99.5%, Merck, Germany) was added to the medium as the sole carbon source; agar-agar (15 g/L; Merck, Germany) was used as a thickening agent only in agar-plate technique.

### 2.3. Inoculum Preparation and Monitoring

Appropriate microbial cultures are often obtained from petrol polluted stations [24]. The microorganisms were isolated from the area of a site contaminated by petroleum products, located in south-western of Iran where has been contaminated by petroleum for at least 100 years. A microbial inoculum able to biodegrade toluene was enriched in the laboratory using batch methods as described in Wolicka et al. [21].

Microbial densities in petrol polluted soil, BV, AIBV experiments, and enriched microbial cultures were measured as the number of colony-forming units (CFU) per gram dry soil, using the agar-plate technique. Moreover, cell density in enriched microbial cultures was determined using optical density measurements via Spectrophotometer (Milton Roy Spectronic 20D, USA) at 600 nm.

### 2.4. Extraction Method

Toluene was extracted from the soil samples with diethylether as a low boiling solvent. Then 1 g of soil sample was added and mixed with 5 mL of diethylether in liquid-tight glass tubes (Schot, Germany) and was vortexed at maximum speed and centrifuged at 6000 rpm for 1and 5 min, respectively. The recovery of this extraction method was 92.8–94.9 percent.

### 2.5. Analytical Measurements

The concentration of toluene in the soil was quantified by a gas chromatography equipped with flame ionization detector (Agilent GC, 7890A, Netherland). The GC-FID procedure was optimized as follows.

The amount of 1 *μ*L of extracted liquid sample was injected into the instrument. Helium with flow rate of 1.11 mL/min was used as the carrier gas and N_2_ with flow rate of 30 mL/min as the makeup gas. Air at 300 mL/min and H_2_ at 30 mL/min were used as flame gases. The characteristic of GC column was Agilent 19091S-433: 30 m × 250 *μ*m × 0.25 *μ*m. The temperatures of the oven, injector, and detector were held fixed at 150, 210, and 250°C, respectively. Toluene quantification was determined by a previously prepared calibration curve with correlation coefficient of 0.999. Toluene showed a retention time of 9.29 min under the experimental condition.

### 2.6. Experimental Setup

**Figure 2:**
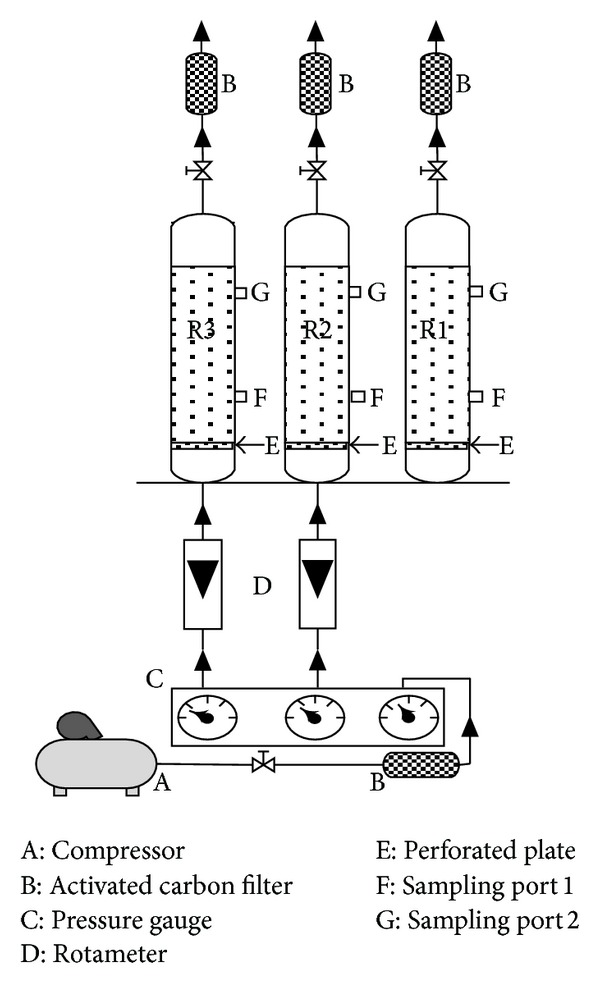
Experimental setup for BV, SVE, and AIBV [reactor 1 (R1): sandy soil + inoculum/MM, reactor 2 (R2): sandy soil + inoculum/MM + Air injection, reactor 3 (R3): sandysoil + sterilized water + air injection].

Figure 2 shows the main component of the experimental setup. Three soil columns (29 cm in length with a 7.29 cm i.d.) were used for the experiments. Two soil sampling ports were located on side of each reactor to obtain soil samples in the column. At the 5 cm height of the column, a perforated stainless steel plate was inserted to maintain the soil and distribution of the inlet gas uniformly. One kg test soil with an initial toluene concentration of 2 mg/g and adjusted moisture content (with sterilized water or inoculum/MM with ratio of 1/10 by volume) was cautiously and rapidly placed into the column. Finally, three activated carbon filters were used to absorb the toluene exiting from columns.

### 2.7. Selection of Optimal Extraction Method

**Figure 3:**
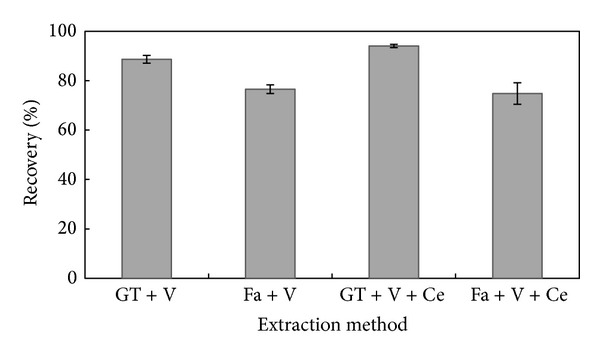
Percent recoveries in four extraction methods (GT: glass tube; V: vortex; Fa: falcon tube; Ce: centrifuge).

For desorption of BTEX, low boiling point solvents such as carbon disulfide, dichloromethane, and acetonitrile are commonly used [25]. Diethylether is one of the low boiling point solvents, that is, employed chemical desorbent in many studies [14, 26–28]. However, in this study diethylether was used as a solvent for extraction of toluene, but for achieving optimal extraction method, a series of experiments were conducted in triplicate. One gram of soil was placed into a test tube and 2 mg toluene was injected to the soil via Hamilton microliter syringe (Hamilton series no. 7000; Hamilton Co., NV) and mixed with 5 mL of diethylether. As seen in Figure 3, the highest recovery of 94.9% was obtained by glass tube, vortexed at maximum speed for 1 min and then centrifuged at 6000 rpm for 10 min. The reason for this difference (between glass tube and falcon tube) can be attributed to reaction or absorption of toluene by falcon tubes.

### 2.8. Toluene Degrading Bacteria

Average bacterial populations in the original petrol soil were determined at 2.5 × 10^5^ colony forming units per gram of dry weight of soil (CFU/gdw). After several steps of enrichment, 1.5 × 10^8^ CFU/mL toluene degrading population was obtained. The Gram-method staining shows that the enriched bacterial cultures were bacilli, cocci, and coccobacillus bacteria. The results obtained were almost the same as given by Wolicka et al. [21].

## 3. Results and Discussion

### 3.1. The Effect of Air Flow Rate on SVE

**Figure 4:**
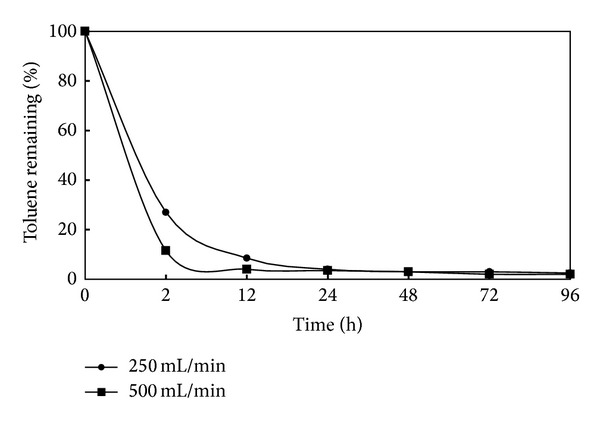
The effect of air flow rate on SVE (moisture content = 10%; initial toluene conc. = 2 mg/g soil).

Figure 4 show the results of the effect of air flow rate on SVE that was performed in R3 column (see Figure 2). The stripping of toluene was quickly increased over the second hour of the air injection. As can be seen in Figure 4, at 250 mL/min flow rate, only 27 percent of toluene remained after a 2 hour air injection. As the increase of air flow rate leads to better stripping [13], only about 12 percent of toluene remained in port 1 and 2, after 2 hours, with 500 mL/min air injection. 24 h after air injection, the differences in toluene removal between the 250 and 500 mL/min is not significant; however, the flow rate of 250 mL/min was used for other experiments.

### 3.2. The Effect of Soil Water Content on SVE

**Figure 5:**
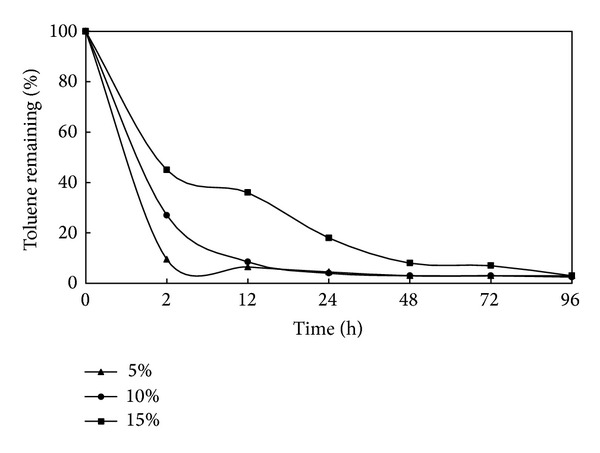
The effect of SWC on SVE (air flow rate = 250 mL/min; initial toluene conc. = 2 mg/g soil).

Like the previous section, this part was also conducted in R3 column. The effects of 5, 10, and 15 percent of SWC on SVE are presented in Figure 5. As it can be seen in this figure, the percent of toluene remaining decreases with reducing the water content. But it can be found that approximately 3 percent of toluene has remained 96 h after air injection. On the other hand 97 percent of toluene has been volatilized in port 1 and 2 on average. The figure also shows that, 2 h after air injection 9, 27, and 45 percent of toluene has remained in sandy soils with 5, 10, and 15 percent water contents, respectively. Some studies showed that, higher rates of volatilization occur at lower levels of soil moisture. Fischer et al. [16] and Poulsen et al. [30] found that increasing SWC led to the decrease of volatilization, since water content diminishes SVE performance due to the occupation of porosity.

### 3.3. Comparison of Three Technologies for Soil Remediation

**Figure 6:**
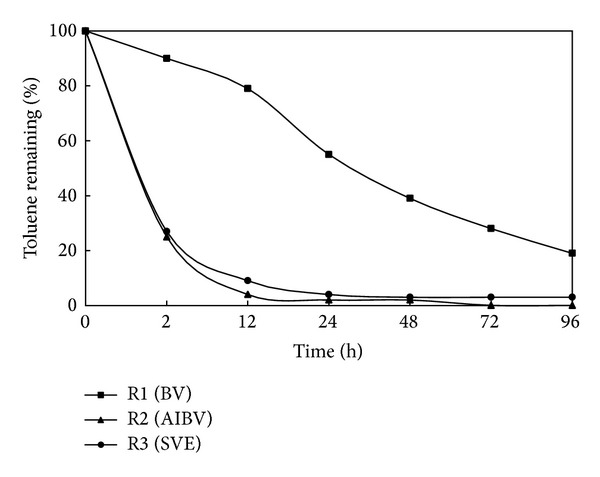
The efficiency of BV, SVE, and AIBV for toluene removal.

For comparison between BV, SVE, and AIBV technologies, three columns in parallel were conducted. A column without air injection (R1) was used as a passive bioventing and control for biodegradation of toluene, and columns R2 (AIBV reactor) and R3 (SVE reactor) were fed by constant flow rate of 250 mL/min.  Figure 6 shows the removal efficiencies of the three technologies. As it can be observed, after a period of 96 h, almost 19, <0.5, and 3 percent of toluene remained in R1, R2, and R3, respectively. As can be seen in Figure 6, in column R2 in the early hours of stripping (by using an air flow rate higher than the lower aeration rate that required for supporting biodegradation) 74% of the contaminant was volatilized and bioventing did not occur. With regard to the remaining percentage of toluene in R2 and R3 after 96 h air injection, the effect of microbial degradation in R2 became evident. Clearly, it was indicated in Figure 6 that SVE alone cannot remediate the polluted soil entirely and BV requires more time for complete bioremediation. Thus, we conclude that AIBV is an efficient biotechnology for soil remediation, especially for treatment of soil contamination as a result of accidental spills of gasoline derivatives.

The important results of some literatures about in situ technologies for vadose zone remediation are presented in Table 2. However, many factors such as porosity, pH, bulk density, sieve size, and organic content of tested soils are different for each study. For example, Hadim et al. [31] showed that remediation in coarser soils are much more rapidly than fine soils. However, the presented studies also confirmed that AIBV is an efficient and reliable biotechnology for soil remediation.

Following remediation timeframe, culturable bacterial population counts were evaluated. So that 1.1 × 10^6^ and 2.3 × 10^5^ CFU/gdw of sandy soil, toluene degrading population, was obtained in R1 and R2 columns, respectively. It should be noted that at least 10^5^ CFU/g of soil microbial population are required for possibility of bioventing [32].

## 4. Conclusions

In this study, the effects of air flow rate and soil water content on the SVE technology were investigated. The results demonstrated that the higher rates of stripping occur at lower levels of soil water content, and higher flow rates result in higher rates of volatilization. Studies of in situ technologies found that BV is an efficient technology to complement SVE. After a 4 day soil decontamination, efficiencies of BV, SVE, and integration of them were 81, 97, and >99.5 percent, respectively. On the other hand, with respect to remediation times and residual concentrations, modification of BV to stripping mode (AIBV) is more efficient than separate remediation tests of BV and SVE.

## Figures and Tables

**Table 1:** Physicochemical properties of sandy soil.

Properties	Values	Methods references
Bulk density (kg/m^3^)	992	ASTM D-854. [22]
Porosity (%)	36	Qin et al. [13]
Sand equivalency (%)	88.7	ASTM D-2419. [22]
pH	7.8	Balba et al. [23]
Size distribution (mesh)	See Figure 1	ASTM C-136. [22]

**Table 2:** In situ remediation tests in literature.

Soil texture	Air flow rate	Type of technology	Initial concentration (mg/g of soil)	Remediation time (day)	Efficiency (%)	Reference(s)
Sand	40 cm^3^/cm^2^·h	AIBV	4 mg/g	11	99	Malina et al. [17]
Sand	40 cm^3^/cm^2^·h	SVE	4 mg/g	24	99	Malina et al. [17]
Qurtz sand	10 mL/min	BV	0.812 mg/g	23.3	30	Sui et al. [29]
Natural soil	—	BV	2 mg/g	5	91	Magalhães et al. [14]
			14 mg/g	5	82	
Natural soil	0.13 L/min	AIBV	2 mg/g	5	99	Magalhães et al. [14]
			14 mg/g	5	99	
Sand	—	BV	2 mg/g	4	81	This study
Sand	0.25 L/min	SVE	2 mg/g	4	97	This study
Sand	0.25 L/min	AIBV	2 mg/g	4	>99.5	This study

—: Without air injection.
